# Sumoylation-deficient Prdx6 gains protective function by amplifying enzymatic activity and stability and escapes oxidative stress-induced aberrant Sumoylation

**DOI:** 10.1038/cddis.2016.424

**Published:** 2017-01-05

**Authors:** Bhavana Chhunchha, Eri Kubo, Nigar Fatma, Dhirendra P Singh

**Affiliations:** 1 Department of Ophthalmology and Visual Sciences, University of Nebraska Medical Center, Omaha, NE, USA; 2 Department of Ophthalmology, Kanazawa Medical University, Kanazawa, Ishikawa, Japan

## Abstract

Aberrant Sumoylation of protein(s) in response to oxidative stress or during aging is known to be involved in etiopathogenesis of many diseases. Upon oxidative stress, Peroxiredoxin (Prdx) 6 is aberrantly Sumoylated by Sumo1, resulting in loss of functions and cell death. We identified lysines (K) 122 and 142 as the major Sumo1 conjugation sites in Prdx6. Intriguingly, the mutant Prdx6 K122/142 R (arginine) gained protective efficacy, increasing in abundance and promoting glutathione (GSH) peroxidase and acidic calcium-independent phospholipase A_2_ (aiPLA_2_) activities. Using lens epithelial cells derived from targeted inactivation of *Prdx6*^−/−^ gene and relative enzymatic and stability assays, we discovered dramatic increases in GSH-peroxidase (30%) and aiPLA_2_ (37%) activities and stability in the K122/142 R mutant, suggesting Sumo1 destabilized Prdx6 integrity. *Prdx6*^−/−^LECs with EGFP-Sumo1 transduced or co-expressed with mutant TAT-HA-Prdx6K122/142 R or pGFP-Prdx6K122/142 R were highly resistant to oxidative stress, demonstrating mutant protein escaped and interrupted the Prdx6 aberrant Sumoylation-mediated cell death pathway. Mutational analysis of functional sites showed that both peroxidase and PLA_2_ active sites were necessary for mutant Prdx6 function, and that Prdx6 phosphorylation (at T177 residue) was essential for optimum PLA_2_ activity. Our work reveals the involvement of oxidative stress-induced aberrant Sumoylation in dysregulation of Prdx6 function. Mutant Prdx6 at its Sumo1 sites escapes and abates this adverse process by maintaining its integrity and gaining function. We propose that the K122/142R mutant of Prdx6 in the form of a TAT-fusion protein may be an easily applicable intervention for pathobiology of cells related to aberrant Sumoylation signaling in aging or oxidative stress.

Maintaining cellular integrity in the face of diverse causes and effects of oxidative stress is a challenge for cells. Oxidative load may determine the activation threshold of antioxidant-survival pathways, and the cell machinery used to alleviate reactive oxygen species (ROS) and stabilize redox potential. The defensive response is regulated through antioxidant defense systems comprising of antioxidant proteins such as superoxide dismutase, catalase, GSH peroxidase and, importantly, Peroxiredoxin 6 (Prdx6), a member of a relatively newly defined family peroxiredoxin.^1, 2, 3, 4^ However, among their many activities, ROS can induce survival or death signaling depending upon their level of cellular concentration.^5^ The proteins can have differential sensitivity and vulnerability to ROS-driven oxidative stress-evoked modifications like Sumoylation, phosphorylation and acetylation.^6, 7^ Oxidative stress-induced aberrant protein modifications have been implicated in the etiology and progression of many human diseases.^8, 9, 10^

Prdx6 exerts its protective function through glutathione peroxidase and aiPLA_2_ activities.^1, 2, 3, 4^ Prdx6 has a single-conserved cysteine residue, known as 1-cysteine (C).^2^ Prdx6 differs from other members of the Prdx family in having both glutathione peroxidase and PLA_2_ activities. A catalytic triad (S32, H26 and D140) is the active site for its PLA_2_ activity and C47 is responsible for GSH peroxidase activity.^2^ Prdx6 is highly expressed in the brain, eye and lung.^2, 8, 11^ It is predominantly localized in the cytoplasm, but is also localized in lysosome, lamellar body, plasma membrane, endoplasmic reticulum, mitochondria and cerebral fluid.^4, 12^ These observations underscore Prdx6's biological importance.^1^ In earlier studies,^8, 13^ we showed that Prdx6 expression is significantly reduced in LECs during oxidative stress. In addition, we reported that aging lenses or lenses/LECs facing oxidative stress display reduced expression of Prdx6 and are susceptible to stressors-induced cell death and lens opacity, and extrinsic application of Prdx6 reverse the injurious process.^1, 8, 13^ In above scenario, we argue that physiological expression level is crucial for Prdx6's biological activity.

Sumos are important post-translational modifiers involved in regulation of various cellular processes by affecting proteins functions,^8, 14^ as many proteins, including Prdx6, are modified by Sumo1 and aberrantly Sumoylated in response to oxidative stress.^8, 15^ Generally, Sumo conjugation occurs in nuclear proteins; however, several cytoplasmic Sumo conjugates have been identified, including Prdx6.^8, 16^ Sumoylation occurs predominantly at a core consensus motif in substrate proteins (Ψ-K-X-[D/E], where Ψ is any large hydrophobic residue (I, V or L), K is target lysine, X is any residue and D/E is aspartate or glutamate).^17^ Recently, an extended consensus motif for Sumo binding was found^18, 19^ and was termed a non-consensus motif for Sumo binding.^8^ Moreover, aberrant Sumoylation signaling has been shown to be a cause of initiation and progression of various diseases including cancer, heart failure, diabetes and pathogenic inflammations caused by infectious agents.^20, 21^ ROS can modulate the process of Sumoylation by affecting the activation of conjugation and deconjugation enzymes.^10, 22^ During oxidative stress, Sumoylation levels have been found to be altered in several proteins, such as HIPK2,^23^ TP53INP1,^24^ Prdx6 and LEDGF.^8, 25^ Furthermore, the crosstalk between Sumoylation and other post-translational modifications including ubiquitination has been well documented.^26, 27^ Sumoylation and ubiquitination can act either cooperatively or independently and thereby determine the fate of proteins and the future of cell integrity.^28, 29^

Prdx6 is aberrantly Sumoylated by Sumo1 during oxidative stress, losing its protective function. We posited that with disruption of Sumo1 site(s), Prdx6 may retain or augment its activity. This hypothesis is supported by the literature showing natural occurrence of several protective gene mutations in animals and humans.^30, 31^ Towards our goal of current study, we identified Sumoylation motif(s) of Prdx6 and determined contribution of each motif(s) in Prdx6 Sumoylation status. We found that Prdx6 is Sumoylated at K122 and K142 residues. Intriguingly, Sumoylation-deficient Prdx6K122/142 R displayed increased enzymatic activities and stability and provided enhanced protection of LECs against oxidative stress and adverse Sumoylation. Discovery of a protective mutant of Prdx6 should provide a foundation for useful strategies for configuring proteins to enhance their protective efficacy and stability.

## Results

### Prdx6 is deSumoylated by Senp1 in hLECs

**Figure 1:**
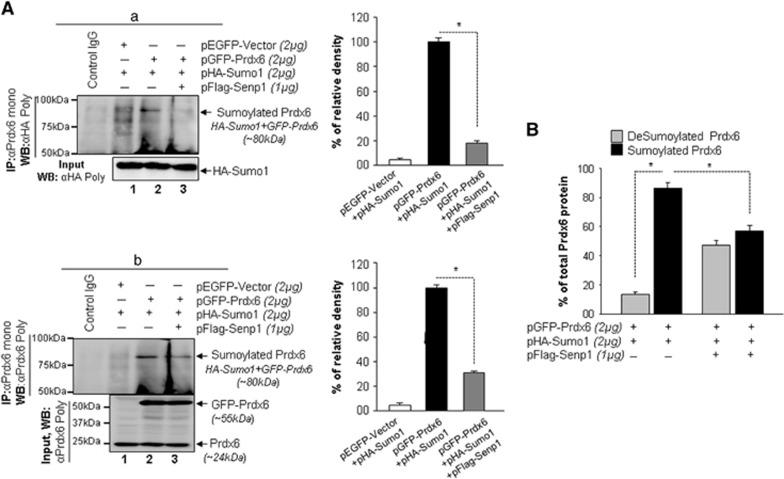
DeSumoylation of Prdx6 required Sumo-specific protease 1 (Senp1) in hLECs *in vivo*. (**A**) Sumoylation and deSumoylation in hLECs by IP assay. LECs were transiently transfected with pCMV (cytomegalovirus)-HA-Sumo1 (pHA-Sumo1) and/or pFlag-Senp1 to express fusion proteins along with pEGFP (enhanced green fluorescent protein)-Vector or pGFP-Prdx6 as shown. 48 h later equal amounts of proteins in cell lysates were used for IP of Prdx6 with Prdx6 monoclonal Ab. Input and IP samples were immunoblotted (western blotted; WB) with anti-HA (A, a) or anti-Prdx6 (A, b) polyclonal Abs and visualized. Results showed a single-exogenous Sumoylated band at ~80 kDa in pHA-Sumo1 plus pGFP-Prdx6 transfected cells (lane 2) and a significant reduction in the Sumoylated band in cells transfected with Senp1 (lane 3) recognized by both anti-HA (A, a) and anti-Prdx6 (A, b) Ab. These results suggest that cotransfection of senp1 led to partial or complete abolishment of Prdx6 Sumoylation. Right panels show densitometric analysis of protein band showing percent Prdx6 Sumoylation. The data represent mean±SD from three independent experiments (*P<0.001). (**B**) Sumo1-Prdx6/ELISA verified deSumoylation of Prdx6 by Senp1. As mentioned in (**a**) extracts isolated from transfectants as indicated and equal amounts of proteins were used for assay as described in 'Materials and methods' section. Total Prdx6 and Sumoylated Prdx6 were determined, and deSumoylated Prdx6 was achieved by subtracting Sumoylated Prdx6 from total Prdx6 protein. The data represents the mean±SD from three independent experiments (*P<0.001)

We investigated if Prdx6 is deSumoylated by Senp1. Cell lysates from hLECs co-transfected with either pEGFP-Vector plus pHA-Sumo1, pGFP-Prdx6 plus pHA-Sumo1 or pGFP-Prdx6 plus pHA-Sumo1 plus pFlag-Senp1 were processes for *in vivo* deSumoylation assays. As shown in Figure 1, a Sumoylated Prdx6 band was detected (Figure 1A; ~80 kDa, lane 2) in pGFP-Prdx6 plus pHA-Sumo1 transfected cells. The Sumoylated Prdx6 band diminished/ablated in pGFP-Prdx6 plus pHA-Sumo1 in the presence of pFlag-Senp1 (Figure 1A; lane 3). Next, we carried out Sandwich/Sumo1-ELISA assay as indicated (Figure 1B) to determine Sumoylated and deSumoylated forms of Prdx6 protein. Cell lysates from transfectants with pGFP-Prdx6 plus pHA-Sumo1 showed ~15% deSumoylated and ~85% Sumoylated forms of Prdx6. In contrast, transfectants with pFlag-Senp1 showed a dramatic shift from Sumoylated to deSumoylated status (~43%). Collectively, Figure 1 showed that Senp1 was responsible for Prdx6 deSumoylation.

### Mutation within Sumoylation motif did not alter localization patterns, and lysines 122 and 142 were major Sumoylation sites in Prdx6

**Figure 2:**
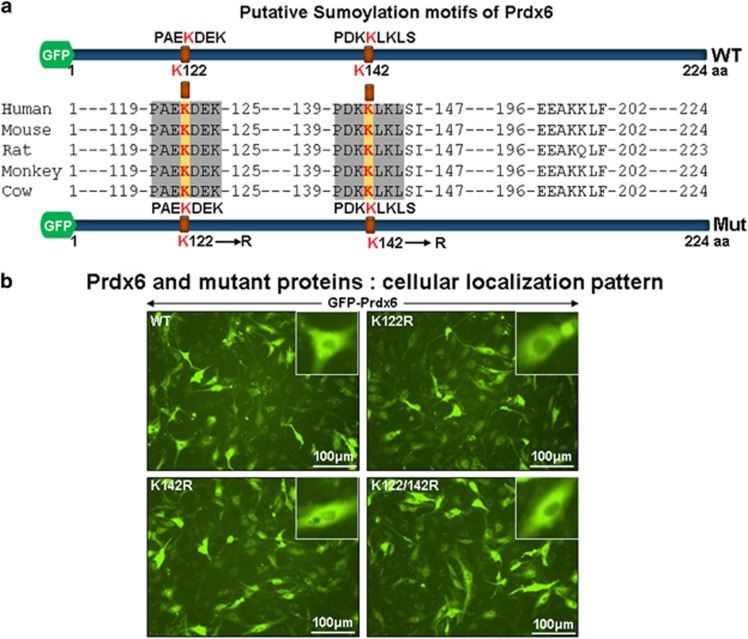
Disruption of Sumo1 sites K122/142 R did not alter cellular localization of Prdx6. (**a**) Schematic diagram of the evolutionary conserved Sumo1 motifs of Prdx6 spotted by SUMOplot and Prdx6 mutants fused to GFP plasmid as shown. SUMOplot, a web-based software program (http://sumosp.biocuckoo.org/online.php), was used to examine Sumo1 conjugation motif. Sequence alignment of human, mouse, rat, monkey and cow Prdx6 protein was conducted to identify evolutionary conserved Sumoylation motif (ClustalW). Lysine (K) residues are indicated in red bold letters. (**b**) Localization of WT and K122R or K142 R or K122/142 R mutant of Prdx6 fused to GFP plasmid. To examine if putative Sumoylation motifs K122 or K142 in Prdx6 protein predicted by Web-based analysis is indeed responsible for Sumo1 conjugation, K residue was changed to R by using SDM and tested for localization pattern for each plasmid. Immunofluorescence images showing localization of Prdx6 and its mutant forms K122R, K142 R and K122/142 R; cells transfected with pGFP-Prdx6WT (WT, upper left panel), mutant pGFP-Prdx6K122R (K122R, upper right panel), pGFP-Prdx6K142R (K142R, lower right panel), pGFP-Prdx6K122/142R (K122/142R, lower right panel) and fluorescence images of live cells were recorded after 24 h of transfection under inverted fluorescence microscope (Nikon Eclipse Ti-U)

Sequence analysis using a SUMOsp2.0 (http://sumosp.biocuckoo.org/archive/prediction.php^32^ and ClustalW programs identified two major putative non-consensus and evolutionary conserved Sumo1 motifs, lysine(K)122 (PAEKDEK) and K142 (PDKKLKL) (Figure 2a) in Prdx6 protein. To ascertain if K122 and/or K142 are indeed Sumoylation motif of Prdx6, we mutated K to arginine(R), generating three Prdx6 mutants at K122R, K142R and K122/142 R (both sites) and examined their subcellular localization by expressing them in hLECs as indicated (Figure 2b). Fluorescence images showed that mutants of Prdx6 were predominantly localized in cytosol and were indistinguishable from Prdx6WT as shown in Figure 2. Next we tested whether overexpression of Sumo1 altered Prdx6 localization. Transfectants with Sumo1 revealed similar localization pattern of Prdx6 as shown (data not shown).

**Figure 3:**
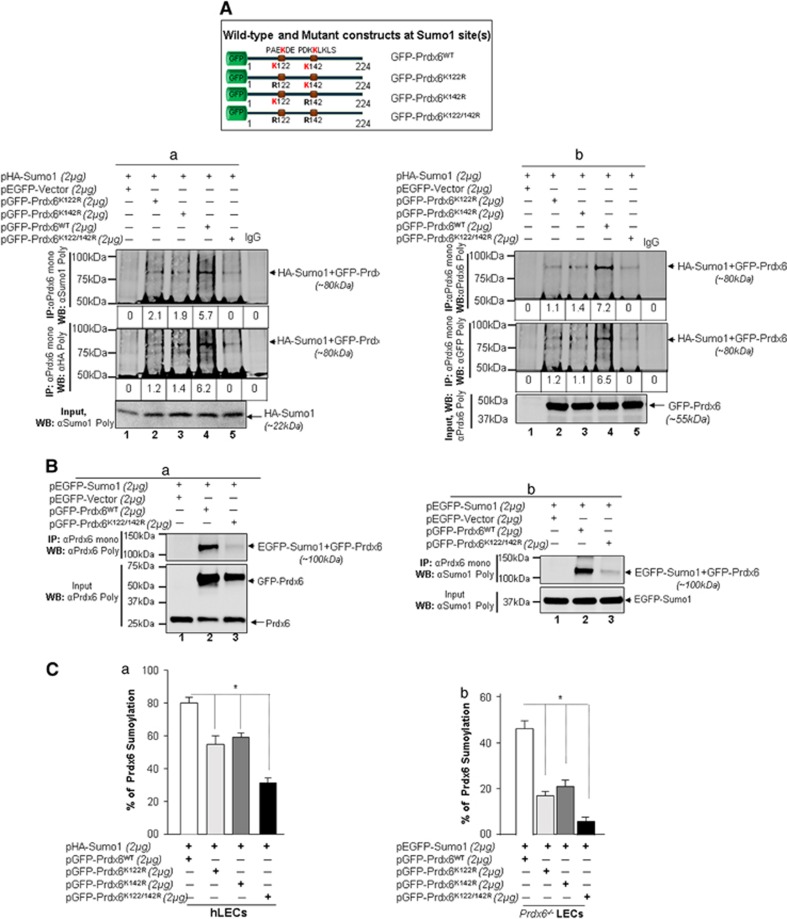
Sumo1 was conjugated to lysine K122 and K142 of Prdx6 *in vivo*. (**A**) Top panel, a diagrammatic illustration of Prdx6WT and its mutant plasmids. hLECs were transfected with pHA-Sumo1 along with pEGFP-Vector, pGFP-Prdx6, pGFP-Prdx6K122R, pGFP-Prdx6K142 R or pGFP-Prdx6K122/142 R plasmids. Cell lysates containing equal amounts of proteins were processed for IP using anti-Prdx6 monoclonal Ab and immunoblotted with anti-Sumo1, anti-HA (**A**, **a**), anti-Prdx6 or anti-GFP (**A**, **b**) polyclonal antibodies as described in 'Materials and methods' section. Input, visualized with anti-Sumo1 and anti-Prdx6 antibodies as shown. IP with Prdx6 monoclonal antibody shows a single-exogenous Sumoylated band at ~80 kDa (lane 4). pHA-Sumo1 co-transfected with pEGFP-Vector or pGFP-Prdx6K122/142 R does not show a Sumoylated band (Lane 5), whereas pHA-Sumo1 co-transfected with pGFP-Prdx6K122R or pGFP-Prdx6K142 R shows reduced Prdx6 Sumoylation. Data indicate both K122 and K142 in Prdx6 sumo1 motifs were targets for Sumo1 modification. (**B**) Confirmation of *in vivo* Sumoylation of Prdx6 at lysine K122 and K142. hLECs were transfected with pEGFP-Sumo1 along with Prdx6WT or its mutant K122/142 R (mutated at both sites) plasmid linked to GFP or pEGFP-Vector as indicated. Prdx6 was immunoprecipitated from cell lysates containing equal amount of proteins, and its Sumoylation was measured with anti-Prdx6 polyclonal antibody (**B**, **a**) and antibody specific to Sumo1 (**B**, **b**) as indicated. Cell lysates were prepared and subjected to IP using anti-Prdx6 monoclonal antibody. IP with Prdx6 monoclonal antibody shows single-exogenous Sumoylated band at ~100 kDa (lane 2, pEGFP-Sumo1+GFP-Prdx6). No Sumoylation band could be detected in cell extracts of pEGFP-Sumo1+pEGFP-Vector or pEGFP-Sumo1+pGFP-Prdx6K122/142 R linked GFP transfected cells (**B**, **a** and **b**; lanes 1 and 3) (**C**) Assessment of conjugation efficiency of Sumoylation motifs of Prdx6 and its mutants to Sumo1 in hLECs and *Prdx6*^−/−^LECs. (**C**,**a**) Conjugation efficiency of Prdx6 and its mutants mutated at only one and both sumo1-binding motifs to Sumo1. hLECs were transfected with pHA-Sumo1 along with pGFP-Prdx6 or its mutants K122R or K142 R or K122/142 R fused to GFP plasmid as shown. Total cell lysates containing equal amount of proteins were processed for Sumo1-ELISA assays to assess the relative efficiency of Sumoylation of Prdx6 and its mutant proteins. The data represent mean±SD from three independent experiments (*P<0.001). (**C**,**b**) Extent of Sumoylation of Prdx6WT and its mutants *in vivo*. *Prdx6*^−/−^LECs were co-transfected with pEGFP-Sumo1 with pGFP-Prdx6WT, pGFP-Prdx6K122R, pGFP-Prdx6K142R, pGFP-Prdx6K122/142R as indicated. Cell lysates consisting of equal amounts of proteins were processed by Sumo1-ELISA assays as described in 'Materials and methods' and as described earlier. Sumoylated content of Prdx6WT and its mutant proteins are presented in percentage. The data represent mean±SD from three independent experiments (*P<0.001)

Next we determined whether predicted Sumoylation sites are indeed Sumoylated, we overexpressed hLECs with pHA-Sumo1 along with pEGFP-vector or GFP-Prdx6WT or its mutants and processed for immunoprecipitation (IP) with antibodies indicated. As shown in Figures 3A and B, IP products when immunoblotted with anti-Sumo1, anti-HA or anti-Prdx6 and anti-GFP antibodies revealed a discrete slower migrating band of HA-Sumo1 plus pGFP-Prdx6 (Figure 3A, ~80 kDa, lane 4). In contrast, mutants GFP-Prdx6K122R or GFP-Prdx6K142R or GFP-Prdx6K122/142 R did not reveal any significant detectable protein bands with any of antibodies indicated (Figure 3Aa and Figure 3Ab, upper and middle panel, lane 5), demonstrating that GFP-Prdx6K122/142 R was not Sumoylated. However, we did observe a very faint Sumoylated Prdx6 band of pGFP-Prdx6K122R or pGFP-Prdx6K142R with indicated antibodies (Figure 3A, ~80 kDa, lanes2 and 3), suggesting that both sites contributed in Prdx6 Sumoylation status. To avoid any artefactual effects, we performed the Sumoylation experiments with EGFP-Sumo1 along with pGFP-Prdx6WT or pGFP-Prdx6K122/142 R and immunoblotted with anti-Prdx6 (Figure 3Ba) and anti-Sumo1 (Figure 3Bb) antibodies. As shown in Figure 3B, a Sumoylated band of pGFP-Prdx6 plus pEGFP-Sumo1 (Figure 3B, ~100 kDa, lane 2) could be observed, whereas no Sumoylated protein band of pGFP-Prdx6K122/142 R could be visible (Figure 3B, lane 3), confirming that Prdx6K122 and 142 are two major Sumoylation sites in Prdx6.

We next investigated relative conjugation efficiency of Sumoylation motifs of Prdx6 to Sumo1 by using Sumo1-ELISA.^8, 25^ Cell lysates from hLECs transfected with pHA-Sumo1 with pGFP-Prdx6 or its mutant plasmids were processed for assay. As shown in Figure 3Ca, transfectants with pHA-Sumo1 along with Prdx6 mutated at a one site showed approximately 38% reduced Sumoylation, whereas mutant K122/142 R showed further ~65% reduction in Sumoylation status (Figure 3Ca). Results revealed that both Sumoylation motifs of Prdx6 had almost equal efficiency of Sumoylation. Furthermore, we also evaluated the extent of Sumoylation of extrinsically expressed Prdx6WT and its mutant plasmids in *Prdx6*^−/−^ LECs. Cell lysates from *Prdx6*^−/−^ LECs overexpressing pEGFP-Sumo1 along with pGFP-Prdx6 or its mutant plasmids as indicated. Notably, K122R and K142R showed 60 and 55% reduced Sumoylation in compared with Prdx6WT, respectively. The value of Sumoylation status was dramatically decreased in the case of mutant K122/142 R as shown in Figure 3Cb.

### Prdx6K122/142 R gained protective potential for rescuing cells from oxidative and aberrant Sumoylation stresses

**Figure 4:**
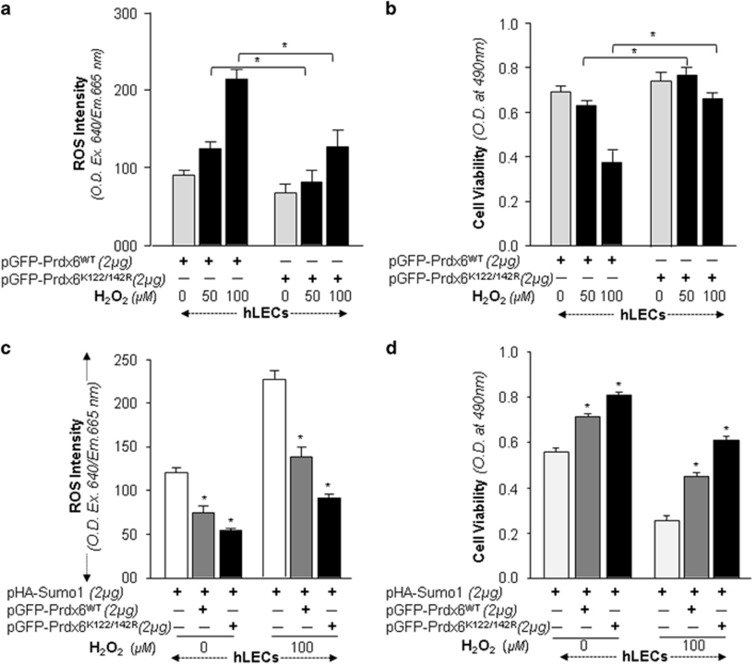
Increased protective efficiency of Sumoylation-deficient Prdx6K122/142R compared with Prdx6WT protein. **a** and **b**, hLECs were transfected with either pGFP-Prdx6 or pGFP-Prdx6K122/142R and then exposed to different concentrations of H_2_O_2_. After 8 h of H_2_O_2_ exposure, ROS intensity was quantified with CellROX deep red reagent (**a**), and 24 h later viability of cells was analyzed by MTS assay (**b**) as indicated. Histogram values represent mean±SD of three independent experiments (*P<0.001). **c** and **d**, hLECs were transfected with pHA-Sumo1 along with either pEGFP-Vector (open bar), pGFP-Prdx6 (gray bar) or pGFP-Prdx6K122/142 R (black bar), and then exposed to oxidative stress. ROS intensity (**c**) and cell viability (**d**) are presented as histograms. Values represent mean±SD of three independent experiments (*P<0.001). Sumoylation-deficient Prdx6K122/142 R (black bar) showed significantly higher protection and reduced ROS production, indicating that mutant Prdx6K122/142 R was more effective at protecting cells from oxidative stress-Sumoylation-mediated insults

Next we asked whether Prdx6K122/142 R would have greater efficacy in protecting cells. hLECs overexpressing pGFP-Prdx6WT or pGFP-Prdx6K122/142 R were exposed to H_2_O_2_ as indicated. GFP-Prdx6K122/142 R transfected cells showed significantly reduced ROS and increased protection (Figure 4) compared with GFP-Prdx6WT as shown by ROS and cell viability assays. Also, we measured the synergistic effect of Sumo1 and oxidative stress on protective efficacy of Prdx6K122/142 R in rescuing cells. The experiments were similar as above, using cells overexpressing Sumo1. When assayed for ROS and cell viability, the transfectants bearing Prdx6K122/142 R were highly efficient in reducing ROS (Figure 4c), and were more resistant to oxidative and Sumo1-induced insults (Figure 4d). Collectively, data suggest that Prdx6K122/142 R rescued the cells by blunting aberrant Sumoylation and oxidative stresses.

### TAT-HA-Prdx6K122/142 R internalized in cells and provided enhanced protection against aberrant Sumoylation and oxidative stresses

**Figure 5:**
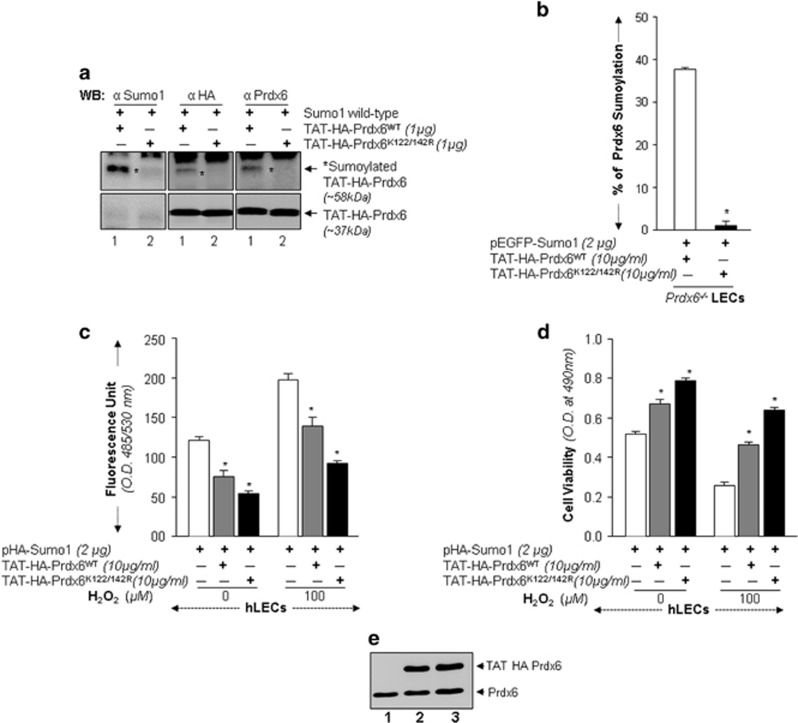
Sumoylation-deficient Prdx6K122/142 R linked to transduction protein domain (TAT) internalized in cells and exerted enhanced protective activity against oxidative stress and Sumo1 overexpression. (**a** and **b**) Confirmation of Sumoylation of mutant TAT-HA-Prdx6K122/142 R and Prdx6WT *in vitro* and *in vivo*. The *in vitro* Sumoylation assay was performed according to the manufacturer's protocol. Briefly, a combination of E1 enzyme, E2 (Ubc9) enzyme, Sumo1WT protein and recombinant Prdx6 protein (TAT-HA-Prdx6) WT or its mutant at K122/142 R were mixed with 20* μ*l reaction mixture containing Sumoylation buffer, as described in 'Materials and methods'. Reaction products were immunoblotted using anti-Sumo1, anti-HA and anti-Prdx6 polyclonal antibodies as indicated. Sumoylation of recombinant Prdx6WT protein was detected, as shown in figure, lane 1 (* denotes the Sumoylation band). In contrast, His-tagged Prdx6 mutated at K122/142 R did not reveal any detectable band (lane 2). (**b**) Sumoylation status of TAT-HA-Prdx6 and its mutants transduced into *Prdx6-*deficient LECs *in vivo*. *Prdx6*^−/−^LECs were transfected with pEGFP-Sumo1, and the transfectants were transduced with TAT-HA-Prdx6 or its mutant TAT-HA-Prdx6K122/142 R as indicated. Cell lysates containing equal amounts of proteins were processed for Sumo1-ELISA assay using anti-HA and antibody specific to Sumo1 as stated in the 'Materials and methods' section. Sumoylated content of Prdx6WT and its mutants proteins are presented as percentages. The data represent mean±SD from three independent experiments (*P<0.001). (**c** and **d**) LECs transduced with Sumoylation-deficient protein, TAT-HA-Prdx6K122/142 R showed higher resistance to oxidative stress-Sumo1 induced damage than did Prdx6WT. hLECs overexpressing pHA-Sumo1 were pretreated with TAT-HA-Prdx6 or TAT-HA-Prdx6K122/142 R and then exposed to H_2_O_2_. Results of ROS (**c**) obtained from H2-DCF-DA dye, and cell survival (**d**) from MTS assay showed that delivery of TAT-HA-Prdx6K122/142 R to cells significantly enhanced protection by efficiently removing ROS. The data represent the mean±SD from three independent experiments (*P<0.001). (**e**) Transduction of TAT-HA-Prdx6 into cells. An aliquot of 10 *μ*g/ml recombinant protein was added to culture media and transduction of TAT-HA-Prdx6 (lane 2) and TAT-HA-Prdx6K122/142 R (lane 3) was assessed using western analysis by anti-Prdx6 antibody

At first, we checked whether recombinant Prdx6 retained the properties of Sumoylation and mutant Prdx6 could serve as Sumoylation-deficient Prdx6 protein. Figure 5a, *in vitro* and Figure 5b, *in vivo* Sumoylation assays^16, 25^ showed that TAT-HA-Prdx6 was Sumoylated at K122 and K142 as observed. Figure 5a shows a Sumoylated Prdx6 band (~58 kDa) and was recognized by antibodies indicated (Figure 5a, lane 1). No detectable Sumoylated band was identified with TAT-HA-Prdx6K122/142 R (Figure 5a, lane 2). Next, we tested whether TAT-HA-Prdx6 or its mutants K122/142 R internalized in cells and thereby retained the Sumo1-binding sites. Sumo1-ELISA showed a dramatic reduction in Sumoylation of mutant TAT-HA-Prdx6K122/142 R compared with Prdx6WT as shown in Figure 5b.

Next, to explore how Sumoylation-deficient Prdx6 might be deliverable, we utilized TAT-linked-Prdx6 and tested its protective efficacy. Cells overexpressing Sumo1 were transduced either with TAT-HA-Prdx6WT or TAT-HA-Prdx6K122/142 R proteins as shown and submitted to oxidative stress. Cells transduced with TAT-HA-Prdx6K122/142 R showed significantly reduced ROS (Figure 5c) and increased cell viability (Figure 5d). Collectively, Figures 5c–e show that the Prdx6K122/142 R efficiently transduced in cells (Figure 5e) and augmented cytoprotection against aberrant Sumoylation and oxidative stresses.

### Sumoylation-deficient mutant Prdx6K122/142 R increased cellular stability

**Figure 6:**
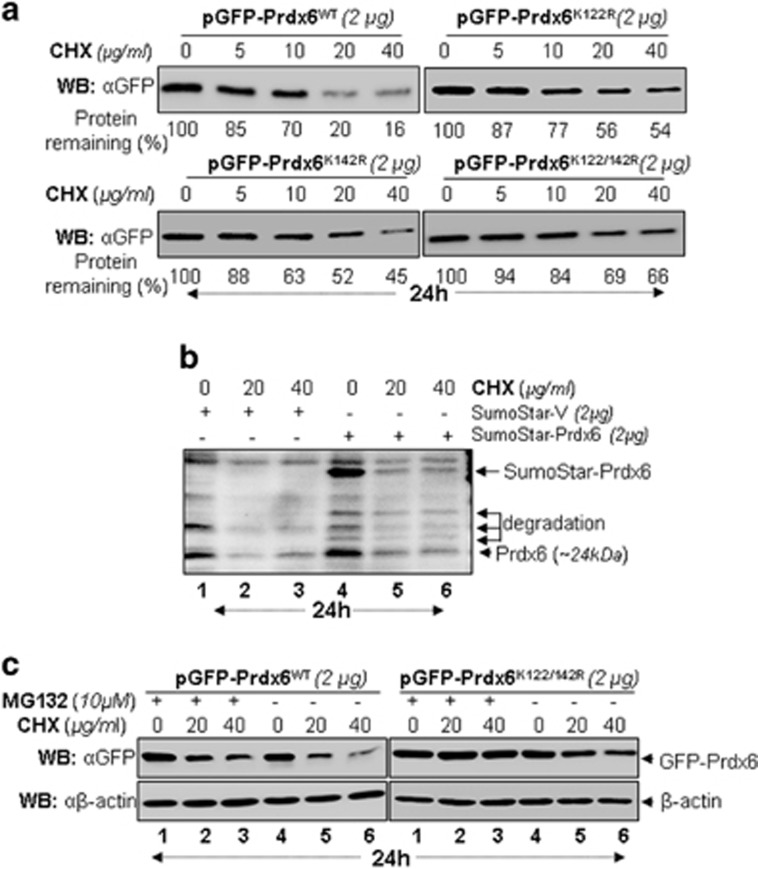
Sumoylation-deficient mutant Prdx6K122/142 R displayed increased steady state levels compared with Prdx6WT. (**a**) Relative protein stability of WT and single or double mutants of Prdx6. hLECs were transiently transfected with pGFP-Prdx6WT or its mutants, pGFP-Prdx6K122R, pGFP-Prdx6K142 R or pGFP-Prdx6K122/142R. After 48 h, the transfectants were treated with different concentrations of CHX for 24 h as indicated. Total lysates with equal amounts of proteins were western blotted (WB) with anti-GFP antibody. The percentage of WT or mutants of Prdx6 protein remaining following the CHX, translational inhibitor treatment is indicated below each protein band based upon densitometry quantitation. (**b**) Sumo1-induced degradation of Prdx6. hLECs were transiently transfected with Sumo-Star-Vector or Sumo-Star-Prdx6 as described in 'Materials and methods'. 48 h later cells were treated with 20 or 40 *μ*g/ml CHX and incubated for 24 h. Total cell lysates with equal amounts of proteins were resolved onto SDS-PAGE and immunoblotted with anti-Prdx6 antibody. (**c**) MG132, the specific proteasome inhibitor treatment supported Sumoylation-mediated Prdx6 degradation via the ubiquitin-proteasome pathway. hLECs were transiently transfected with pGFP-Prdx6WT and/or pGFP-Prdx6K122/142 R as indicated. 48 h later the transfectants were treated with different concentrations of CHX after 3 h of DMSO or 10*μ*M MG132 (lanes 1-3) treatment as indicated. Total cell lysates with equal amounts of proteins were WB with anti-GFP antibody

To test if Sumoylation would affect Prdx6 stability, we analyzed the cellular stability of Prdx6WT and its mutants by dismissing *de novo* protein synthesis with cycloheximide (CHX), a translational inhibitor. Cells transiently transfected with GFP-Prdx6WT or its mutants were treated with CHX as indicated. As shown in Figure 6a, Prdx6 mutants at Sumoylation sites were more stable than the Prdx6WT; the remaining protein Prdx6 WT and its mutant forms are shown in percentages under the protein bands based on densitometry quantitation analysis. We found that cellular abundance of mutants K122R or K142R or K122/142 R proteins significantly higher than GFP-Prdx6WT protein at 20 *μ*g/ml and 40 *μ*g/ml (Figure 6a), suggesting that it is likely that Sumoylation mediates Prdx6 degradation. In this scenario we posited that an observed decline in Prdx6 abundance in cells could be due to changes in Sumoylated Prdx6 stability (Figure 6a). Hence, we next examined whether Sumo1 conjugation to Prdx6 affects its stability. Sumoylation is a highly dynamical process. Hence, it has been difficult to detect Sumo-mediated degradation of specific protein. To overcome this issue, we used a Sumo fusion strategy that has been successfully used in the past with proteins.^33, 34^ Toward this, we used Sumo1-Prdx6 fusion plasmid to transfect cells as described in the 'Materials and methods' section. Cell lysates from transfectants with Vector or pM-Sumo1-Prdx6 followed by different concentrations of CHX treatment were immunoblotted with antibody as indicated. Figure 6b shows increased degradation in pM-Sumo-Prdx6, suggesting indeed Sumo1 is involved in Prdx6 destabilization.

Next, we examined whether Sumoylation mediates Prdx6 degradation through proteasomal pathway, cells overexpressing GFP-Prdx6WT or GFP-Prdx6K122/142 R were treated with 10 *μ*M MG132, an inhibitor of proteasome pathway, in the presence/absence of CHX.^35, 36, 37^ Immunoblotting of lysates with Prdx6 antibody showed that the cellular abundance of both forms increased in cells treated with MG132; however, the levels of mutant Prdx6 was significantly higher (Figure 6c), demonstrating the role of Sumoylation in Prdx6 degradation.

### Disruption of Sumoylation motif K122/142 R in Prdx6 promoted PLA_2_ and GSH peroxidase activities

**Figure 7:**
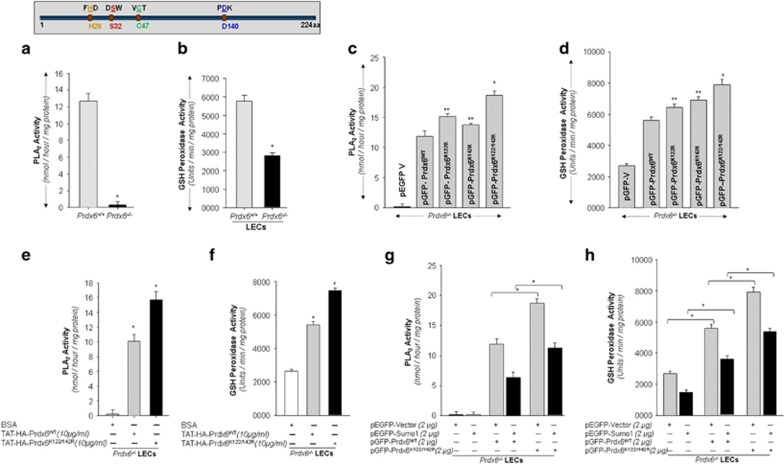
(**a** and **b**). *Prdx6*-deficient LECs displayed insignificantly low levels of Phospholipase A_2_ as well as lower GSH peroxidase activities compared with *Prdx6*^+/+^. *Prdx6*^+/+^ and *Prdx6*^−/−^ LECs cultured in identical conditions as described in 'Materials and methods'. Cells were harvested and total extracts containing equal amounts of proteins were processed to measure PLA_2_ (**a**) and glutathione peroxidase activity (**b**) following the company's protocols. Black bars show significantly reduced PLA_2_ and GSH peroxidase activities in *Prdx6*-deficient cells. The data represent the mean±SD from three independent experiments (*P<0.001). Upper panel, a schematic illustration of active sites responsible for PLA_2_ (S32/H26/D140) and GSH peroxidase (C47) activities. (**c** and **d**). Disruption of Sumoylation motif K122/142 R in Prdx6 protein promoted PLA_2_ and glutathione peroxidase activities. *Prdx6*^−/−^LECs were transfected with pEGFP-Vector, pGFP-Prdx6WT and its mutants, pGFP-Prdx6K122R, pGFP-Prdx6K142 R and pGFP-Prdx6K122/142R fused to GFP plasmids. After 48 h, total lysates containing equal amounts of proteins were processed for PLA_2_ (**c**) and GSH peroxidase (**d**) activities through Enzchek PLA_2_ and GSH peroxidase assay kits (Invitrogen), respectively. (Prdx6WT *versus* mutants; *P<0.001; **P<0.05). (**e** and **f**). Recombinant mutant Prdx6K122/142 R protein had increased PLA_2_ and GSH peroxidase activities compared with Prdx6WT. *Prdx6*^−/−^LECs were transduced with TAT-HA-Prdx6 and its mutant TAT-HA-Prdx6K122/142 R. After 24 h, total protein was isolated and assays were performed for PLA_2_ (**e**) and Glutathione peroxidase (**f**) activities as described in ‘Materials and methods' section. The data represent the mean±SD from three independent experiments (*P<0.001). (**g** and **h**). Sumo1 overexpression diminished phospholipase PLA_2_ and GSH peroxidase activities. *Prdx6*^−/−^LECs were co-expressed with pEGFP-Sumo1 along with pEGFP-Vector or pGFP-Prdx6WT or its mutant K122/142 R, 48 h later total cell lysates containing equal amounts of proteins were utilized to measure PLA_2_ (**g**) and glutathione peroxidase (**h**) activities; results are presented as nmol/min/mg protein and units/min/mg protein, respectively. The data represent the mean±SD from three independent experiments (*P<0.001)

We examined whether mutation at Sumoylation motifs influences Prdx6 activity. At first, we confirmed PLA_2_ and GSH peroxidase activities of Prdx6 as reported by others previously.^2, 4^ We used *Prdx6*^+/+^ and *Prdx6*^−/−^ LECs and analyzed PLA_2_ (Figure 7a) and GSH peroxidase (Figure 7b) activities. PLA_2_ activity was undetectable in *Prdx6*^−/−^ LECs, but did display 45% GSH peroxidase activity. These results were similar to earlier reports.^2, 4^ Next, we measure the effect of Sumoylation on Prdx6's enzymatic activity. *Prdx6*^−/−^ cells transfected with pGFP-Prdx6WT or its sumo1 mutants. Cell lysates from transfectants were processed to measure PLA_2_ (Figure 7c) and GSH-peroxidase (Figure 7d) activities. It was surprising to observe that Prdx6K122/142R displayed significantly increased PLA_2_ (Figure 7c) and GSH-peroxidase activities (Figure 7d) compared with Prdx6WT. Next we tested whether TAT-HA-Prdx6 and its mutants internalized in cells, had similar PLA_2_ and GSH peroxidase activities as observed, we transduced TAT-HA-Prdx6 and its mutant recombinant protein in *Prdx6*^−/−^ LECs (Figure 7). As expected, mutant Prdx6 recombinant proteins had higher GSH peroxidase and PLA_2_ activities (Figures 7e and f).

Next we tested if Sumo1 influences Prdx6's activities, cell lysates from *Prdx6*^−/−^ expressing pEGFP-Sumo1 or pEGFP-Vector along with pGFP-Prdx6WT or pGFP-Prdx6K122/142 R were analyzed for enzymatic activity as shown in Figure 7. In the presence of Sumo1, GSH peroxidase and PLA_2_ activities were reduced in Prdx6WT. To our surprise, we also observed a reduction in PLA_2_ and GSH peroxidase activities of Prdx6K122/142 R, though the activities were still significantly higher. However, we are unable to explain how overexpression of Sumo1 dysregulated the Prdx6 active sites.

### Contribution of PLA_2_, S32/H26/D140 and GSH peroxidase, C47 sites to Prdx6's cytoprotective activity

**Figure 8:**
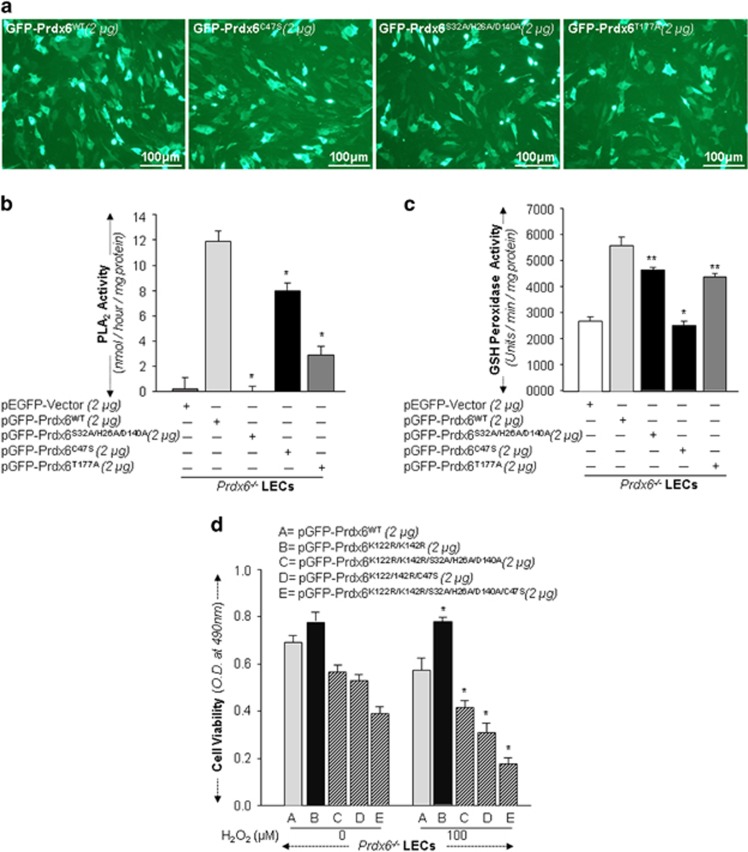
Effect of mutation at sites responsible for Prdx6's PLA_2_ and GSH peroxidase activities and contribution of Phosphorylation site. (**a**) Photomicrograph shows the transfection efficiency of plasmids used. *Prdx6*^−/−^cells were transfected with plasmids containing mutation at sites responsible for PLA_2_ (S32/ H26/ D140 to A) or GSH-peroxidase (C47S) activity and/or phosphorylation (T177A). PLA_2_ (**b**) and glutathione peroxidase (**c**) activities were assayed as described in the 'Material and methods' section. Serine (S)32- Histidine (H)26- Aspartic acid (D)140, a catalytic triad important for Prdx6 Phospholipase A_2_ (PLA_2_) activity, and cysteine (C) 47, responsible for Prdx6 antioxidant activity. Prdx6 phosphorylate at Threonine (T) 177. (WT *versus* mutant; *P<0.001, **P<0.05). (**d**) Loss of protective activity of Prdx6 or mutant Prdx6K122/142 R with mutation at PLA_2_, S32A/H26A/D140A and GSH peroxidase sites, C47S. *Prdx6*^−/−^cells were transfected with pGFP-Prdx6 or its mutant constructs as indicated. These transfectants were submitted to H_2_O_2_-induced oxidative stress, and 24 h later cell viability was measured. (WT *versus* mutants; *P<0.001)

Prdx6 is known to achieve its bifunctional protective activity through PLA_2_ and GSH peroxidase activities.^1, 2, 4^ We determined whether mutation at each active site of Prdx6 affects its cytoprotective potential. We mutated cysteine(C)47 to serine(S)47 and PLA_2_ site, the catalytic triad, serine(S) 32-histidine (H) 26-aspartic acid(D)140 to alanine(A) using site-directed-mutagenesis (SDM). To examine the effect of phosphorylation on Prdx6 activity, we mutated threonine (T)177, a phosphorylation site of Prdx6 to A177.^2, 4^ *Prdx6*^−/−^ deficient LECs transfected with pEGFP-vector or GFP-Prdx6 and Prdx6 plasmid containing mutation as indicated were processed to assess GSH peroxidase and PLA_2_ activities. As shown in Figure 8b, we found significantly higher PLA_2_ activity in Prdx6WT, whereas there was no detectable activity in Prdx6 mutated at PLA_2_ sites (S32A/H26A/D140A). Notably, we also observed reductions in PLA_2_ activity (~34%) in Prdx6C47S and 76% in Prdx6T177A. These results suggest that for full PLA_2_ activity both C47 and phosphorylation T177 sites are essential. In a parallel experiment, we found significantly higher GSH peroxidase activity in GFP-Prdx6, which was significantly reduced in Prdx6C47S. Furthermore, reductions of 20% and 25% in GSH peroxidase activity were observed in PLA_2_ mutant and GFP-Prdx6T177A transfected cells, respectively. Taken together, our results demonstrate that PLA_2_ along with phosphorylation and peroxidase sites all are essential to the protective potential of Prdx6.^4, 38^

Since mutation at Sumo1 sites of Prdx6 may alter activity by altering its confirmation, next we tested whether mutant Prdx6K122/142 R active sites behave functionally similar to Prdx6WT. Using *Prdx6*^−/−^ LECs, we conducted cell viability experiments to define relative protective activity of Prdx6WT (Figure 8d, as A) and Prdx6K122/142 R (as B) or Prdx6 K122/142 R having mutation at PLA_2_ (as C) or C47S (as D) or both active sites, (as E) in response to oxidative stress.^8^ Transfectants with plasmid ‘A' and ‘B' displayed increased resistance against oxidative stress, in contrast, transfectants with ‘C' or ‘D' or E (Figure 8d). Taken together, these results indicate that disruption at Sumoylation motifs enhanced protective potential by increasing enzymatic activities of Prdx6. The lack of protection is similar to that of Prdx6WT with mutations at the same sites.

## Discussion

Oxidative stress alters the Sumoylation status of nuclear as well as cytoplasmic proteins, and thereby alters the function and stability of gene products.^8, 16, 25, 39, 40^ In the present study, we identified novel Sumoylation site(s) of Prdx6 that involves conjugation of the Sumo1 to K122 and K142 and, notably, both motifs are evolutionarily well conserved (Figures 2 and 3). By using biochemical and mutational assays, we provide evidence that Sumoylation-deficient Prdx6K122/142 R achieved a cellular steady state and greater protective activity in comparison with Prdx6WT. Analysis of Sumo1-binding motifs of Prdx6 revealed that they did not belong to classical core-Sumo motif; Sumo1 bound to non-consensus motif as shown in Figure 2. Recently, several new Sumo targets have been identified having an extended Sumo consensus motifs^19, 41^ and these targets are both nuclear and nonnuclear proteins.^42, 43^ Previously we showed that Prdx6 is aberrantly Sumoylated during oxidative stress, losing its protective activity and stability.^8^ Our current study revealed that mutation of Prdx6 at Sumo1 sites dramatically enhanced its protective potential and stability (Figures 4 and 6). The steady physiological state and function of Sumoylated proteins depend upon a balance between Sumoylation/deSumoylation processes in cellular background. During aging or oxidative stress Senp1 is dimerized and becomes inactive^8, 34, 44^ leading to an increase of free Sumos. Sumoylation process has been shown to be highly sensitive to internal/external stimulus, and these stimuli can modulate the status of proteins due to changes in Sumos expression and Sumoylation processes.^8, 16, 39, 41^ In earlier reports, we demonstrated that when cells overexpressing Sumo1 along with Prdx6 are subjected to oxidative stress, they become more vulnerable to cellular insults. In this work, we examined the influence of Sumo1 overexpression on stability and activity of Sumoylation-deficient Prdx6K122/142 R during oxidative stress. Figures 4 and 5 disclose that in fact Sumo1-deficient Prdx6 became more efficacious in protecting cells from oxidative stress. From the lens of therapeutic intervention, we also tested the protective potential of transduction domain-linked-Prdx6WT and Prdx6K122/142 R. We found that TAT-HA-Prdx6K122/142 R was more efficacious in rescuing cells from oxidative stress-driven aberrant Sumoylation signaling.^3, 45^ This experiment provided a proof of concept that Prdx6 or proteins with protective mutation can be utilized to combat disorders related to oxidative stress or aberrant Sumoylation signaling. TAT-linked protein can internalize in cells/tissues and has been found to be biologically active.^3, 46^ Thus, in both normal physiological condition and oxidative stress, mutant Prdx6K122/142 R can enhance cell survival by blocking exaggerated oxidative damage of cells. It would be worth to mention that several earlier cell culture-based experiments have examined the biological functions and mechanisms of action of chemicals/biomolecules, and those have found the same functions or activities *in vivo*, but with different concentrations and regimens.^47, 48^ Thus we think that our study should clarify the modulated protective activity of mutant Prdx6 mutated at Sumo1 sites in protecting cells against oxidative stress, and that these findings can be tested for translational outcomes *in vivo*.^1, 3, 13, 49, 50^

Moreover, in the current study, we used Prdx6-deficient lens epithelial cells (LECs) derived from Prdx6 knock-out mice to deliver mutant Prdx6. A careful examination of these cells revealed that these cells were indistinguishable from controls transfected or transduced with empty vector or inactive protein, suggesting that mutation does not adversely affect LECs integrity, but rather enhances their survival against stress. Furthermore, lysine residue(s) is a target for various modifications, like methylation, acetylation, ubiquitination, Sumoylation, and so on, and these post-translational modifications are an important event in gene regulation and function.^51^ Nevertheless, bioinformatics analyses revealed that these two sites, K122/142 in Prdx6 were a plausible and putative target for Sumo1 modification, and we found that indeed both sites are Sumoylated.^8^ Importantly, how these two residues, K122 and K142, have been (specifically) selected for Sumo1 conjugation during evolution is a very cumbersome to understand and dictate; we posit that this could be happen through random or spontaneous selection process of gene. However, our aim in the current study was how to escape adverse effects of aberrant Sumoylation signaling that causes dysregulation of Prdx6 leading to cell death. At this juncture we postulated that Sumoylation-deficient Prdx6 should be the best strategy for avoiding stress-induced aberrant Sumoylation signaling. These mutations (K122/142 R) could be beneficial, neutral or harmful for cells, tissues or organisms, as the mutations do not recognize what the cells require for the best. Fortunately, we found that mutant Prdx6 mutated at Sumoylation site(s) had greater protective potential. This postulation is supported by published studies showing the occurrence of several protective or deleterious gene mutations linked to disease states.^30, 31, 52^

Recent reports reveal that proteins of different backgrounds can differ for substrate specificity to be Sumoylated.^22, 25, 53^ Magnitude of oxidative stress is crucial to both deSumoylation/Sumoylation of proteins.^25, 34, 44, 54, 55^ In the case of Prdx6, increased Sumoylation jeopardized its function by reducing its stability and enzymatic activity. Sumoylation is analogous, and mechanistically very similar, to the ubiquitination pathway and involves E1, E2 and E3 enzymes.^56^ But the ultimate biological effects of both are different. Sumo1 binding to lysine residue within Sumoylation motif can change protein stability, localization pattern and many functions.^57, 58^ Conversely, ubiquitination by binding of ubiquitin chain to lysine results in rapid degradation through the 26 S proteasomal pathway. In the present work, we found that increased Sumoylation of Prdx6 destabilized it (Figure 6), and perturbed the genetically allotted functions (Figures 4 and 5) in redox-active cells.^8^ Figure 6 shows that the cellular steady state of Sumoylation-deficient Prdx6 is greater compared with Prdx6WT. Indeed, our experimentation disclosed that Sumoylation induced Prdx6 degradation through proteasome -pathway, as was evident from experiments with MG132, an inhibitor for proteasomal pathway. Sumoylation is known to be involved in degradation as well as stabilization of target proteins.^37, 59^ Ubiquitin conjugation site(s) in Prdx6 have not been defined as yet, bioinformatics analyses revealed that a putative ubiquitination site in Prdx6 might be lysine 192 (personal observation). However, detailed study is warranted to delineate the role of Sumo1 in modulation of the ubiquitination process in context to Prdx6 degradation during oxidative stress.

In examining the cause for increased protective activity of Sumoylation-deficient Prdx6, we found a significant increase in GSH peroxidase and PLA_2_ activities compared with Prdx6WT (Figure 7). However, we could not be able to explain this surprising outcomes how the activities of Prdx6K122/142 R are increased. We surmise that conformational changes due to mutation at K122R and K142R may provide better interface or additional configuration for Prdx6 interactions and activities. It is worth to mention that we also observed a reduction in GSH peroxidase and PLA_2_ activities in transfectants overexpressing Sumo1, mostly influencing PLA_2_ activity, but activities of both were higher than that of Prdx6WT (Figures 7g and h). However, how Sumo1 interferes active sites of Prdx6 requires investigation. Moreover, mutation enhances the activities of many proteins, possibly reflecting the evolutionary process of nature. Several protective, modulating, functional genes have been discovered in animals as well as in humans. These genetic mutations may be either beneficial or harmful for cells, depending upon cell background. However, in many gene products, mutation modulates their activities, and such proteins justify the occurrence and continuation of the evolution process for survival of cells/tissues/species in adverse environments and fatal disease states. Importantly, recombinant proteins like insulin, growth hormones, interferon, erythroprotein and others have been successfully used for therapeutic purposes.^60^ On the basis of our previous finding and current work and coupled with other published works, our observations suggest that enhancing protective functions of Prdx6 by mutation at Sumo1 sites may offer a novel therapeutic strategy for diseases related to oxidative stress and its associated aberrant Sumoylation-mediated pathogenic signaling. Furthermore, this study shows that ROS-induced aberrant Sumoylation of Prdx6 dramatically decreases the protein's stability and function, leading to cell death, a finding which may be relevant to understanding the cause of many diseases.

## Materials and Methods

### Cell culture

Human LECs (hLECs) (a kind gift of Dr. Venkat N. Reddy, Eye Research Institute, Oakland University, Rochester, MI, USA) were maintained in Dulbecco's modified Eagle's medium (Invitrogen, Carlsbad, CA, USA) with 15% fetal bovine serum (Atlanta Biologicals, Inc., Flowery Branch, GA, USA), 100 *μ*g/ml streptomycin and 100 *μ*g/ml penicillin in 5% CO_2_ environment at 37 °C as described previously.^8^ Cells were harvested and cultured in 96, 24, 48 or 6 well plates and 100 mm petri dishes according to the requirements of the experiment(s).

### Western blot analysis and antibodies

Total cell lysates were prepared in ice-cold radio IP assay (RIPA) lysis buffer, as described previously. Equal amounts of protein samples were loaded onto 10%, 12% or 4–20% SDS-PAGE gel, immunoblotted onto PVDF membrane (Perkin Elmer, Waltham, MA, USA) using indicated antibodies.^8^ The following antibodies were used: Prdx6 monoclonal (Lab Frontier, Seoul, Korea), Prdx6 monoclonal (IP grade, Abcam, Cambridge, MA, USA), Prdx6 polyclonal (Santa Cruz Biotechnologies, Santa Cruz, CA, USA), Prdx6 rabbit polyclonal (LS-B8135, LS Bio, Seattle, WA, USA), Prdx6 monolonal (LS-B6255, LS Bio), HA polyclonal (ab9110, Abcam), Sumo1 monoclonal (Santa Cruz Biotechnologies, Santa Cruz, CA, USA), Sumo1 polyclonal (Santa Cruz Biotechnologies, Santa Cruz, CA, USA), Sumo1 polyclonal (Active motif), GFP monoclonal (IP grade, Santa Cruz Biotechnologies, Santa Cruz, CA, USA), GFP polyclonal (Invitrogen) and Senp1 monoclonal (Santa Cruz Biotechnologies, Santa Cruz, CA, USA). To ascertain comparative expression and equal loading of the protein samples, the membrane stained earlier was stripped and re-probed with *β*-actin antibody or other antibodies shown.

### Construction of DNA plasmid

A full length of Sumo1 cDNA was cloned into pEGFP-C1 vector.^25^ The coding region of Sumo1 was amplified by PCR from human lens cDNA library using forward (Fw) (5′-CCGTCGACATGTCTGACCAGGAG-3′) and reverse primer (Rv) (5′-TCGGATCCGTTTTGAACACCACA-3′) with restriction enzyme sites, *Sal*I and *BamH*I and ligated into pEGFP-vector. pFlag-Senp1 was a generous gift from Dr. E. Yeh (University of Texas M.D. Anderson Cancer Center, Houston, TX, USA). All the transfection experiments were carried out either with Superfactamine Reagent (Invitrogen) or by using the Neon Transfection system (Invitrogen).

### TAT-HA-Prdx6 recombinant protein Purification

A full-length cDNA of Prdx6 from a human LEC cDNA library using Prdx6-specific Forward (5′-GTCGCCATGGCCGGAGGTCTGCTTC-3′ contained *NcoI* site) Reverse (5′-AATTGGCAGCTGACATCCTCTGGCTC-3′) was ligated into a TA-cloning vector (Invitrogen), plasmid consisting cDNA was amplified cloned into a pTAT-HA expression vector at *NcoI* and *EcoRI sites* (a kind gift of Dr. S. F. Dowdy). Wild-type (WT) TAT-HA- Prdx6 was then mutated at K (lysine) 122 R (arginine), K142 R and K122/142R by using SDM kit. Recombinant proteins was purified from transformants (*Escherichia coli* BL21 (DE3)) using QIAexpress Ni-NTA Fast Start kit column (Qiagen Inc., Valencia, CA, USA) as described.^8, 13^ This purified protein can be either used directly for protein Sumoylation, or aliquoted and stored frozen in 10% glycerol at −80 °C for further use.

### *In vitro* and *in vivo* Sumoylation assay

Purified recombinant TAT-HA-Prdx6 or its mutant at K122/142 R protein were incubated with E1, E2 and Sumo1 protein for 3 h at 30 °C according to the manufacturers' protocol (SUMOlink *in vitro* SUMO-1 Kit, Catalog no. 40120, Active Motif, Carlsbad, CA, USA). The reaction was stopped by adding an equal amount of 2 × SDS-PAGE loading buffer and immunoblotted. Sumoylation bands were visualized by anti-Prdx6 or anti-Sumo1 or anti-HA antibody as described previously.^8, 25^

hLECs were co-transfected with pEGFP-Sumo1/pHA-Sumo1 and pEGFP-vector or pGFP-Prdx6 or pGFP-Prdx6K122R or pGFP-Prdx6K142 R or pGFP-Prdx6K122/142R as indicated in figures. After 48 h, total cell lysates were prepared in IP lysis/wash buffer (0.025 M Tris, 0.15 M NaCl, 0.001 M EDTA, 1% NP-40, 5% glycerol, pH7.4 plus 5 *μ*M MG132 and 30*μ*M NEM (N-ethylaleimide) (Pierce Classic IP Kit, Catalog No. 26146, Thermo Scientific, Rockford, IL, USA) as per manufacturer's instructions. Total cell lysates were incubated with indicated antibodies for IP as described previously.^8, 25^ 10% Input and IP samples were immunoblotted by using the indicated antibodies.

### Sandwich- ELISA/ Sumo1-ELISA

A total Prdx6 protein and its Sumoylated form was performed by sandwich-ELISA (enzyme linked immunosorbent assay; Abnova, Taipei City, Taiwan) and EpiQuik *in vivo* universal protein Sumoylation assay kit following the companies' protocols and as described previously.^8^ Briefly, hLECs or *Prdx6*^−/−^ LECs were transfected with plasmids empty vector, Sumo1, Senp1, Prdx6 and its mutant forms (K122R, K142 R and K122/142R) as indicated in the figures. After 48 h, total cell lysates from transfectants containing equal amount of proteins were loaded in ELISA plate well coated with Prdx6 polyclonal antibody followed by incubation with monoclonal anti-Prdx6 antibody. After incubation with goat anti-mouse-HRP conjugated secondary Ab, OPD substrate was used for color development and OD (optical density) was recorded at 450 nm.

Sumoylated Prdx6 was detected in cell extracts from transfectants by using an EpiQuik *in vivo* universal protein Sumoylation assay kit (Epigentek, Farmingdale, NY, USA). In brief, cell extract with equal amount of proteins was added to the strip wells, which were percolated anti-Prdx6 antibody or control IgG. After three washes, anti-Sumo1 antibody was added. Following color development by a Sumo detection system, absorbance was measured at 490 nm using an ELISA plate reader. To obtain deSumoylated form of Prdx6; values of Sumoylated Prdx6 protein was subtracted from total Prdx6 protein and presented as deSumoylated Prdx6.

### Generation and validation of LECs isolated from lenses of *Prdx6*^−/−^ and *Prdx6*^+/+^ mice

All animal experiments followed the recommendations set forth in the Statement for the Use of Animals in Ophthalmic Research by the Association for Research in Vision and Ophthalmology. Animal studies were approved by the University of Nebraska Medical Center, Omaha, NE, USA. LECs isolated from Prdx6-targeted mutants (*Prdx6*^−/−^) and wild-type (*Prdx6*^+/+^) mice were generated and maintained in Dulbecco's Modified Eagle's Medium (Invitrogen) with 10% fetal bovine serum (Atlanta Biologicals, Inc.) as described earlier.^1^ We used *Prdx6*^−/−^ mutant mice which are maintained on fully inbred C57B6 background, and, as controls, wild-type C57B6 inbred mice of the same sex and age (*Prdx6*^+/+^). This minimizes the variation due to genetic background. All animals were maintained under specific pathogen-free conditions in an animal facility. LECs were isolated from mice of identical age, and Western analysis was carried out to confirm the presence of *α*A-crystalline,^1^ a specific marker of LECs. Cells from 3–5 passages were used for the experiments.

### pM-Sumo-Star-Prdx6 Eukaryotic expression construct

To detect the direct effect of Sumo1 conjugation of Prdx6 on Prdx6 integrity, we constructed Sumo1-Prdx6 fusion plasmid by cloning a full cDNA of Prdx6 into pM-Sumo-Star eukaryotic expression vector between *BsmB1* and *XhoI* sites (Life Sensors, Malvern, PA, USA) using Prdx6 Forward (5′-CGTCTCTAGGTATGCCCGGAGGTCTGCTTCTCG-3′) and Reverse (5′-CTCGAGTCATCACAGCACCAGCTTCTCCAA-3′) primers containing *BsmB1* and *XhoI* restriction sites, respectively.^8^ Plasmid was amplified, sequenced and was used for transfection assays.

### Site-Directed mutagenesis

PCR-based site-directed mutagenesis was carried out with the QuikChange site-directed mutagenesis kit (Invitrogen), following the company's protocol. SDM primers used were as follows.

**Prdx6 Lysine (K) 122 to Arginine (R) 122, (K122R)**

Forward: 5′-GGCATGCTGGATCCAGCAGAGAGGGATGAAAAGGGC-3′

Reverse: 5′-GCCCTTTTCATCCCTCTCTGCTGGATCCAGCATGCC-3′

**Prdx6 K142 to R142, (K142R)**

Forward: 5′ -GGTCCTGATAAGCGGCTGAAGCTGTCTATCCTCTACCC-3′

Forward: 5′-GGGTAGAGGATAGACAGCTTCAGCCGTTATCAGGACC-3′

**Prdx6 Histidine (H) 26 to Alanine (A) 26, (H26A)**

Forward: 5′- CGGCCGCATCCGTTTCGCCGACTTTCTGGGAGACTC -3′

Reverse: 5′- GAGTCTCCCAGAAAGTCGGCGAAACGGATGCGGCCG -3′

**Prdx6 Serine (S) 32 to A32, (S32A)**

Forward: 5′- CCACGACTTTCTGGGAGACGCATGGGGCATTCTCTTCTCC -3′

Reverse: 5′- GGAGAAGAGAATGCCCCATGCGTCTCCCAGAAAGTCGTGG -3′

**Prdx6 Aspartic acid (D) 140 to A140, (D140A)**

Forward: 5′-GTGGTGTTTGTTTTTGGTCCTGCAAAGAAGCTGAAGCTGTCTATCC-3′

Reverse: 5′-GGATAGACAGCTTCAGCTTCTTTGCAGGACCAAAAACAAACACCAC -3′

**Prdx6 Threonine (T) 177 to A177 (T177A)**

Forward: 5′- GCAGAAAAAAGGGTTGCCGCCCCAGTTGATTGGAAGGATGGGG-3′

Reverse: 5′-CCCCATCCTTCCAATCAACTGGGGCGGCAAGGGTTTTTTCTGC -3′

**Prdx6 Cysteine (C) 47 to Serine (S) 47, (C47S)**

Forward: 5′-CTTTACCCCAGTGTCCACCACAGAGCTTGGCAGAGC-3′

Reverse: 5′-GCTCTGCCAAGCTCTGTGGTGGACACTGGGGTAAAG-3′

### Cycloheximide, a translational blocker and/or MG132, proteasome inhibitor treatment

To inhibit translation/ protein synthesis, transfected cells as indicated were treated with 0–40 *μ*g/ml CHX for 24 h, and Proteasomal pathway was blocked by using 10 *μ*M MG132. All inhibitors were purchased from Sigma-Aldrich. In case of combination of inhibitor treatment and MG132, cells were first subjected to proteasomal inhibitor for 3 h followed by translational inhibitor CHX for further 24 h. On the day of termination of experiment, total cell lysate prepared and immunoblotted with specific antibodies as indicated in figure and legends.

### Quantitation of intracellular ROS level by H2-DCF-DA and CellROX deep red reagent

Intracellular ROS level was measured by use of fluorescent dye dichlorofluorescin diacetate (H2-DCF-DA), a nonpolar compound that is converted into a polar derivative (dichlorofluorescein) by cellular esterase after incorporation into cells.^1^ On the day of the experiment, the medium was replaced with Hank's solution containing 10 mM H2-DCF-DA dye and cells were incubated. Following 30 min later, intracellular fluorescence was detected with excitation at 485 nm and emission at 530 nm by a Spectra Max Gemini EM (Mol. Devices, Sunnyvale, CA, USA).

ROS level were measure according to the company's protocol (CellROX Deep Red Oxidative Stress Reagent, Catalog No. C10422, Thermo Scientific, Carlsbad, CA, USA). In brief, LECs (5 × 10^3^) transfected with GFP-Prdx6 and GFP-Prdx6K122/142 R alone or with HA-Sumo1 cultured in 96-well plate, 48 h later cells were exposed with different concentration of H_2_O_2_. After 8 h, CellROX deep red reagent was added with final concentration of 5*μ*M and cells were incubated at 37°C for 30 min. Media containing CellROX deep red reagent were removed and fixed with 3.7% formaldehyde. After 15 min, fluorescence signal were measured at Ex640 nm/ Em665 nm.

### Cell viability assay

A colorimetric MTS assay (Promega, Madison, WI, USA) was performed as described earlier.^1, 3^ This assay of cellular proliferation/viability uses 3-(4, 5-dimethylthiazol-2-yl)-5-(3-carboxymethoxyphenyl)-2 to 4-sulphophenyl) 2H-tetrazolium salt (MTS). When added to medium containing viable cells, MTS is reduced to a water-soluble formazan salt. The *A*_490 nm_ value was measured after 2 h with an ELISA reader. Results were normalized with absorbance of the untreated control(s).

### Measurement of phospholipase A_2_ (PLA_2_) activity

Phospholipase A2 activity was measured according to the manufacture's protocol (EnzChek Phospholipase A2 kit; E10217, Invitrogen). In brief, LECs transfected with different plasmid constructs were harvested and cell lysates were isolated. Proteins were measured (Bradford method) and normalized with GFP reading. For standard curve, PLA_2_ stock solution (500 units/ml) diluted with 1 × reaction buffer to make different concentration (0–10 units/ml) of PLA_2_. For sample, equal amount of protein were diluted with 1 × PLA_2_ reaction buffer up to 50 *μ*l volume, then 50 *μ*l of the substrate-liposome mix were added to each microplate well containing standards, controls and samples to start the reaction with total volume 100 *μ*l. The fluorescence of each well was measured at Ex485 nm/Em535 nm using microplate reader (DTX 880, Multimode Detector, and Molecular Device) and presented.

### Glutathione peroxidase activity

Glutathione peroxidase activity measure according to manufacturer's protocol (Glutathione Peroxidase activity kit, Cat No. ADI-900-158, Enzo Life Sciences, Farmingdale, NY, USA). In brief, total cell lysate prepared from LECs transfected with different plasmid constructs as indicated. Cell lysates were isolated from each group transfectants and proteins were estimated, equalized and normalized with GFP values. 140 *μ*l of 1 × assay buffer, 20 *μ*l of 10 × reaction buffer and 20 *μ*l glutathione peroxidase, controls and sample were added to 96-well plate then initiated reaction by quickly adding 20 *μ*l of cumene hydroperoxide to each well. OD was measured at absorbance 340 nm every 1 min over a 10–15 min period. OD of blank is subtracted from the standard and sample OD to obtain the net rate of absorbance at 340 nm for the calculation of glutathione peroxidase activity.

### Statistical method

Data are presented as mean±SD of the indicated number of experiments. Data were analyzed by Student's *t*-test when appropriate. A *P*-value of **P<0.050 and *P<0.001 was defined as indicating a statistically significant difference.
